# Satisfaction With Life, Satisfaction With Job, and the Level of Care Rationing Among Polish Nurses—A Cross-Sectional Study

**DOI:** 10.3389/fpsyg.2021.734789

**Published:** 2021-09-28

**Authors:** Aleksandra Kołtuniuk, Izabela Witczak, Agnieszka Młynarska, Karolina Czajor, Izabella Uchmanowicz

**Affiliations:** ^1^Department of Nervous System Diseases, Faculty of Health Sciences, Wroclaw Medical University, Wroclaw, Poland; ^2^Department of Health Care Economics and Quality, Faculty of Health Sciences, Wroclaw Medical University, Wroclaw, Poland; ^3^Department of Gerontology and Geriatric Nursing, School of Health Sciences, Medical University of Silesia, Katowice, Poland; ^4^Department of Ophthalmology, Wroclaw Medical University, Wroclaw, Poland; ^5^Department of Clinical Nursing, Faculty of Health Sciences, Wroclaw Medical University, Wroclaw, Poland

**Keywords:** care rationing, satisfaction with job, satisfaction with life, nurse, care, BERNCA-R

## Abstract

**Background:** Rationing of nursing care is a serious issue that has been widely discussed throughout recent years in many countries. The level of satisfaction with life and of satisfaction with job as the nurse-related factors may significantly affect the level of care rationing.

**Aim:** To assess the rationing of nursing care among the Polish nurses and the impact of nurse-related variables, i.e., satisfaction with life and satisfaction with job on the level of nursing care rationing.

**Materials and Methods:** A cross-sectional study was conducted among 529 Polish registered nurses employing in two University Hospitals. Three self-report scales in the Polish version were used in this study, namely, Basel Extent of Rationing of Nursing Care-revised version (BERNCA-R), Satisfaction with Life Scale (SWLS), and Satisfaction with Work Scale (SWWS).

**Results:** The respondents indicated that the most frequently rationed activity is studying the situation of individual patients and care plans at the beginning of the shift. The least frequently rationed activity indicated by the respondents was adequate hand hygiene. The patient-to-nurse ratio and the level of satisfaction with job are significant independent factors affecting the level of care rationing.

**Conclusions:** The assessment of the level of satisfaction with life and identification of factors affecting this assessment will enable reducing the occurrence of care rationing.

## What is Already Known About the Topic?

- The level of satisfaction with job depending on the attitudes and feelings of the employee toward the performed profession is a factor that affects the rationing of nursing care significantly.
- Satisfied employees are usually more productive and feel stronger attached to their workplace.

It was also demonstrated that the level of satisfaction with job affects the level of satisfaction with provided services among the patients.

## Introduction

Each patient has a right to the high quality of care provided by a competent nursing team regardless of its clinical condition (New Zealand Nurses Organisation, 2014), Numerous studies (Chegini et al., 2020; Dhaini et al., 2020; Friganovic et al., 2020; Gurková et al., 2020; Jaworski et al., 2020; Kalánková et al., 2020; Uchmanowicz et al., 2020b; Zeleníková et al., 2020) performed throughout recent years demonstrated that, regrettably, the nurses fail to perform all activities planned in the nursing process when delivering care to the patient. This phenomenon was described for the first time by Kalisch (2006) while the literature is replete with many terms defining it, i.e., nursing care left undone (Aiken et al., 2001), unfinished nursing care (Sochalski, 2004), missed nursing care (Kalisch, 2006), implicit care rationing (Schubert et al., 2007, 2008), task incompletion (Al-Kandari and Thomas, 2009), unmet nursing care needs (Lucero et al., 2010), care left undone (Ausserhofer et al., 2014), work left undone (Leary et al., 2014), nursing tasks left undone (Bekker et al., 2015), failure to maintain (Bail, 2016), or the unfinished task of nursing care (Kebede et al., 2017). All these define the condition in which the patient is left without adequate nursing care. This may lead to many undesired events, i.e., falls (Schubert et al., 2008; Lucero et al., 2010; Kalisch et al., 2012), bedsores (Schubert et al., 2008), infections (Lucero et al., 2010), and even increased mortality (Schubert et al., 2012).

To prevent rationing, the studies were performed aiming at the identification of the determinants for the level of nursing tasks left undone. These determinants (factors) were divided into patient-related [i.e., age, type of disease, form (severe/chronic), concomitant diseases, diagnosing, and treatment plan], associated with the working environment (i.e., management, interdisciplinary cooperation, autonomy, and responsibility, as well as material resources and staff shortage), and nurse-related (i.e., experience, knowledge, and skills, as well as psychological factors, e.g., professional burnout, satisfaction with life, and satisfaction with job) (Kalisch et al., 2009; Schubert et al., 2013; Bragadóttir et al., 2017; Griffiths et al., 2018; Jaworski et al., 2020; Mandal et al., 2020; Uchmanowicz et al., 2020a).

Satisfaction with life is defined as the state of balance between the current situation and personal expectations. The more our needs are fulfilled, the more we are satisfied with life (Loewe et al., 2014). According to the studies performed to date, nurses are generally satisfied with life; however, there are some areas of life that could be improved (Piotrkowska et al., 2019; Bartosiewicz et al., 2020; Jaworski et al., 2020). The impact on the level of satisfaction with life in nurses is multifactorial and includes age and education level, among others (Piotrkowska et al., 2019; Bartosiewicz et al., 2020). It was also demonstrated that the nurses with a higher level of satisfaction with life tend to ration nursing care less frequently (Jaworski et al., 2020).

Also, the level of satisfaction with job depending on the attitudes and feelings of the employee toward the performed profession is a factor that affects the rationing of nursing care significantly (Jaworski et al., 2020; Uchmanowicz et al., 2020a). Satisfied employees are usually more productive and feel stronger attached to their workplace. It was also demonstrated that the level of satisfaction with job affects the level of satisfaction with provided services among the patients (Janicijevic et al., 2013).

The aim of this study was to assess the phenomenon of care rationing among the Polish nurses and the impact of nurse-related variables, i.e., satisfaction with life and satisfaction with job on the level of rationing of nursing care in the patients hospitalized at the medical treatment wards.

## Materials and Methods

### Setting, Design, and Participants

This study was performed among surgical nurses from two University Hospitals in Poland (in Wrocław and Katowice) between July and December 2019. The criterion for inclusion in this study was full-time employment in the surgical ward for at least 6 months, work in direct patient care in the ward, and consent to participate in this study. The exclusion criterion was incorrect or incomplete completion of the questionnaires or withdrawal of consent to participate in this study. It included 529 respondents. Participation was voluntary, and respondents were completely anonymous. Of note, 89% of the surveys collected were completed, and 529 respondents were included in the analysis. Questionnaires were administered by pencil-and-paper in a packet completed at one time over 15–20 min. The STROBE guidelines (Strengthening the Reporting of Observational Studies in Epidemiology) were followed.

### Ethical Considerations

This study used survey methods. All respondents received information about the study procedure and aim and provided informed consent to participate. Full anonymity was guaranteed. This study used standardized research questionnaires and a survey collecting sociodemographic data. The study protocol was accepted and approved by the Bioethics Committee of Medical University of Silesia (PCN/0022/KB/17/20).

### Qualification Procedure

The inclusion criteria were as follows: work experience of more than 6 months, work at surgical wards, and consent to participate in this study. The exclusion criteria were as follows: work experience under 6 months and lack of consent to participate in this study.

### Research Instruments

For the purpose of this study, the following instruments were used: the BERNCA questionnaire (Schubert et al., 2007) in Polish version (Uchmanowicz et al., 2019a), the Satisfaction with Life Scale (SWLS) (Diener et al., 1985) in Polish version (Juczyński, 2001), the Satisfaction with Job Scale (Zalewska, 2003), and the questionnaire designed by the authors who asked questions about sociodemographic characteristics. Questions included age, marital status, education, specialization, seniority, the number of places of work, and the number of patients being under nurse care at the unit.

### The BERNCA-R Questionnaire

The Basel Extent of Rationing of Nursing Care-revised version (BERNCA-R) questionnaire was created by Schubert et al. (2007). This tool consists of 32 questions concerning necessary activities and tasks in the work of nurses that might potentially not be performed in everyday work due to a shortage of nursing resources. The questionnaire is divided into five dimensions, namely, daily activities, care and support, rehabilitation and education, monitoring and safety, and documentation. Responses are given on a five-point Likert scale (0**—**not required, 1**—**never, 2**—**rarely, 3**—**sometimes, and 4**—**often) and refer to the last seven working days. For each response, the measure of the rationing frequency is the mean score of overall 32 items; the mean rationing score ranged from 0 to 4.0. The Polish version was adopted by Uchmanowicz et al., and Cronbach's alpha for the unidimensional scale was 0.96 (Uchmanowicz et al., 2019a).

### The Satisfaction With Life Scale

The SWLS was developed by Diener et al. (1985), and the Polish-language version used in this research study was developed by Juczyński (2001). It is a five-item scale that measures global cognitive judgments of the life satisfaction of an individual. Participants indicate how much they agree or disagree with each of the five items using a seven-point scale that ranges from 7 “strongly agree” to 1 “strongly disagree.” Scores range from 5 to 35 points**—**the higher the score, the greater the sense of life satisfaction. After standardization of the general results in the sten scale, the sten scores between 1 and 4 are considered low satisfaction, from 7 to 10 as high satisfaction, and 5 and 6 as average satisfaction. Cronbach's alpha for the Polish version is 0.81, and for the original SWLS 0.87.

### The Satisfaction With Work Scale

The Satisfaction with Work Scale (SWWS) was developed in 1991, and the Polish-language version used in this research was developed by Zalewska (2003). It is five-item scale that measures cognitive judgments of the spheres of work of employees. The respondents answer particular statements about their job satisfaction on a seven-point scale (1**—**I definitely do not agree, 2**—**I do not agree, 3**—**I rather do not agree, 4**—**it is difficult to say whether I agree or do not agree, 5**—**I rather agree, 6**—**I agree, and 7**—**I definitely agree). The overall score ranges from 5 to 35, with higher scores indicating higher job satisfaction. Cronbach's alpha for the Polish version is 0.86.

### Statistical Analysis

The BERNCA-R scores in two groups were compared using the Mann-Whitney *U* test. The BERNCA-R scores in three and more groups were compared using the Kruskal-Wallis tests. Upon identifying the statistically significant differences, the *post-hoc* analysis using Dunn's test to identify the statistically significantly differing groups was performed. Correlations between quantitative variables and the BERNCA-R scores were analyzed using the Spearman correlation coefficient. Multifactorial analysis on the effect of independent multiple variables on the BERNCA-R score was performed using the linear regression method. The results are presented in the form of regression model parameter values with 95% CI.

The significance level adopted for analysis is 0.005. Any and all *p*-values below 0.05 are interpreted as demonstrating significant correlations.

The analysis was performed using the R software version 4.0.2, R Foundation, Vienna, Austria.

## Results

### Sociodemographic Characteristics of Respondents

The analysis of research materials demonstrated that the most represented groups among the respondents were the persons aged 40–50 years (41.97%), being in a relationship (73.37%), with secondary vocational education (41.59%). Detailed data are presented in Table 1.

**Table 1:** Characteristics of groups.

**Parameter**		***n***	**Percentage (%)**
Age	23–30 years	60	11.34
	31–40 years	43	8.13
	41–50 years	222	41.97
	>50 years	204	38.56
Seniority	0–5 years	67	12.67
	6–15 years	35	6.62
	16–20 years	41	7.75
	>20 years	386	72.97
Education	MSc. in nursery/obstetrics	83	15.69
	Bachelor in nursery/obstetrics	152	28.73
	Medical high school	74	13.99
	Medical college and “other” education	220	41.59
Specialty	No	370	69.94
	Yes	159	30.06
Number of jobs	One	361	68.24
	Two or more	168	31.76
Patient-to-nurse ratio	1–5 patients	32	6.05
	6–15 patients	300	56.71
	16–25 patients	136	25.71
	>25 patients	61	11.53
Marital status	In a relationship	404	76.37
	Single	125	23.63

### The BERNCA Questionnaire Results

Analysis of data obtained from the BERNCA questionnaire revealed that the mean total score for the studied group was 1.53, which means that the frequency of care rationing by the respondents falls within the range of between “never” and “rarely” (Table 2).

**Table 2:** Total mean scale score: 1.53 (*SD* = 0.79).

**BERNCA-R [score]**									
**Scale range**	**N**	**No data**	**Mean**	**SD**	**Median**	**Min**	**Max**	**Q1**	**Q3**
0–4	529	0	1,53	0,79	1,5	0	4	1,06	1,91

The respondents indicated that the most frequently rationed activities included the following (Table 3):

- studying the situation of individual patients and care plans at the beginning of shift (question 29)**—**(mean 1.95),
- assessment of the needs of newly admitted patients (question 30)**—**(mean 1.93),
- administering prescribed medication or infusion on time (question 23)**—**(mean 1.89),
- setting up care plans for the patients (question 31)**—**(mean 1.89),
- providing emotional or psycho-social support to the patient (question 10)**—**(mean 1.8).

The least frequently rationed activities indicated by the respondents included (Table 3):

- adequate hand hygiene (question 27)**—**(mean 1.08),
- providing the patients with sufficient information on planned tests or therapies (question 12)**—**(mean 1.2),
- restraining of confused patients due to inability to ensure sufficient monitoring (question 20)**—**(mean 1.26),
- applying necessary disinfection measures (question 28)**—**(mean 1.29)
- preparing the patients for planned tests or therapies (25)**—**(mean 1.3).

**Table 3:** Distribution of answers of the BERNCA-R guestionnaire.

**Question/Items**	**There was no need (0) (%)**	**Never (1) (%)**	**Rarely (2) (%)**	**Sometimes (3) (%)**	**Often (4) (%)**	**No responses (%)**	**Mean**
1. Sponge bath	39.32	25.52	15.50	13.99	5.67	0.00	1.21
2. Partial sponge bath	36.48	32.14	17.77	10.78	2.84	0.00	1.11
3. Skin care	25.33	33.08	24.95	12.10	4.54	0.00	1.37
4. Oral hygiene	29.68	28.54	21.55	13.23	6.99	0.00	1.39
5. Dental hygiene	30.62	24.76	22.31	14.93	7.37	0.00	1.44
6. Assist food intake	24.95	33.84	27.79	11.34	2.08	0.00	1.32
7. Mobilization	20.42	22.12	35.35	17.20	4.91	0.00	1.64
8. Change of the position	22.50	29.68	29.68	13.42	4.73	0.00	1.48
9. Change of the bed linen	19.66	24.39	34.59	13.04	8.32	0.00	1.66
10. Emotional and psychological support	17.39	18.34	34.22	20.04	10.02	0.00	1.87
11. Necessary conversation	19.09	17.77	33.46	21.36	8.32	0.00	1.82
12. Information about therapies	17.39	39.13	24.95	14.74	3.78	0.00	1.48
13. Continence training (diapers)	30.06	29.87	19.85	15.50	4.73	0.00	1.35
14. Continence training (insert catheter)	34.22	33.27	16.45	10.59	5.48	0.00	1.2
15. Activating or rehabilitating care	33.65	13.99	15.31	14.74	22.31	0.00	1.78
16. Education and training	27.22	21.55	28.17	15.12	7.94	0.00	1.55
17. Preparation for discharge	21.36	23.82	28.54	19.47	6.81	0.00	1.67
18. Monitoring patients as described by physician	15.12	32.14	27.22	18.34	7.18	0.00	1.7
19. Monitoring patients as the nurse felt necessary	13.80	26.09	33.65	19.28	7.18	0.00	1.8
20. Monitoring of confused patients and use of restrains	40.64	23.06	15.31	12.10	8.88	0.00	1.26
21. Monitoring of confused patients and use of sedatives	34.40	25.33	16.07	16.26	7.94	0.00	1.38
22. Delay in measure because of a physician delay	24.20	34.40	19.66	16.64	5.10	0.00	1.44
23. Administration of medication. infusions	10.96	23.82	37.24	21.17	6.81	0.00	1.89
24. Change of wound dressings	17.20	43.10	24.20	12.29	3.21	0.00	1.41
25. Preparation for test and therapies	13.80	53.88	23.06	6.99	2.27	0.00	1.3
26. Keep patient waiting who rung	15.12	41.59	24.01	13.99	5.29	0.00	1.53
27. Adequate hand hygiene	27.98	46.88	16.82	5.48	2.84	0.00	1.08
28. Necessary disinfection measures	9.83	63.52	17.77	5.67	3.21	0.00	1.29
29. Studying care plans	9.83	23.44	36.67	22.31	7.75	0.00	1.95
30. Assessment of newly admitted patient	9.83	21.17	41.40	21.17	6.43	0.00	1.93
31. Set up care plans	9.26	26.28	37.62	20.04	6.81	0.00	1.89
32. Documentation and evaluation of the care	8.70	28.73	39.32	17.96	5.29	0.00	1.82

### The SWLS Results

The analysis of the obtained data revealed that 224 among 529 of the questionnaire respondents (42.34%) declared a moderate level of satisfaction with life, 175 respondents (33.08%) declared a high level of satisfaction with life, while 130 respondents (24.57%) exhibited a low level of satisfaction with life.

### The SWWS Results

According to the analysis, the mean SWWS score amounted to 18.5 points, which gives 3.7 points per question. Thus, the respondents were neither satisfied nor dissatisfied with their job.

### Impact of Variables on the Level of Rationing of Nursing Care

Analysis of the obtained data demonstrated that the frequency of care rationing was significantly higher:

- among the persons aged 23–30, 31–40, and 41–50 years compared with the persons aged >50 years,
- among the persons with seniority between 0 and 5 years compared with the persons with seniority >20 years,
- in persons serving 6–15 patients compared with the persons serving 16–25 and >25 patients, in whose it was significantly higher than in persons serving 1–5 patients (Table 4).

**Table 4:** Impact of sociodemographic variables on the level of rationing of nursing care.

**Parameter**	**Group**	**BERNCA-R [score]**			***p***
		**Mean ± SD**	**Median**	**Quartiles**	
Age	23–30 years (*N* = 60)—A	1.68 ± 0.73	1.59	1.43–1.91	*p* = 0.006^*^
	31–40 years (*N* = 43)—B	1.7 ± 0.58	1.62	1.34–1.97	
	41–50 years (*N* = 222)—C	1.55 ± 0.8	1.53	1.04–1.91	B.A.C > D
	>50 years (*N* = 204)—D	1.43 ± 0.83	1.38	0.97–1.84	
Seniority	0–5 years (*N* = 67)—A	1.7 ± 0.72	1.59	1.34–1.97	*p* = 0.031^*^
	6–15 years (*N* = 35)—B	1.63 ± 0.52	1.69	1.5–1.88	
	16–20 years (*N* = 41)—C	1.5 ± 0.84	1.53	1.09–1.81	A > D
	>20 years (*N* = 386)—D	1.5 ± 0.82	1.44	1–1.84	
Education	MSc in nursery/obstetrics (*N* = 83)	1.47 ± 0.85	1.5	0.97–1.78	*p* = 0.056
	Bachelor of nursery/obstetrics (*N* = 152)	1.62 ± 0.76	1.56	1.16–1.94	
	Medical high school (*N* = 74)	1.32 ± 0.52	1.46	1.25–1.56	
	Medical college and “other” education (*N* = 220)	1.57 ± 0.86	1.52	0.97–2.06	
Specialty	No (*N* = 370)	1.54 ± 0.78	1.5	1.16–1.87	*p* = 0.66
	Yes (*N* = 159)	1.52 ± 0.82	1.5	1–1.92	
Number of jobs	One (*N* = 361)	1.54 ± 0.79	1.5	1.09–1.84	*p* = 0.903
	Two or more (*N* = 168)	1.52 ± 0.81	1.5	1.03–1.98	
Patient-to-nurse ratio	1–5 patients (*N* = 32)—A	0.96 ± 0.65	0.89	0.52–1.33	*p* < 0.001^*^
	6–15 patients (*N* = 300)—B	1.67 ± 0.84	1.59	1.09–2.06	
	16–25 patients (*N* = 136)—C	1.44 ± 0.62	1.47	1.16–1.69	B > C.D > A
	>25 patients (*N* = 61)—D	1.39 ± 0.77	1.47	0.91–1.78	
Marital status	In a relationship (*N* = 404)	1.49 ± 0.78	1.5	1.02–1.82	*p* = 0.038^*^
	Single (*N* = 125)	1.68 ± 0.82	1.62	1.16–2.03	

* *Statistically significant correlation (p <0.05)*.

It was also demonstrated that the level of satisfaction with life had no significant impact on the level of rationing of nursing care (*r* = −0.06, *p* > 0.05), while the level of satisfaction with job significantly affects the level of rationing (*r* = −0.232, *p* < 0.001). This means that the higher level of satisfaction with the job, the lower level of rationing of nursing care.

The linear regression model revealed that the significant (*p* < 0.05) independent predictors of the BERNCA-R score include the following:

- 6–15 served patients: regression parameter is 0.675, which increases the BERNCA-R score by 0.675 points on average comparing with 1–5 patients,
- 16–25 served patients: regression parameter is 0.466, which increases the BERNCA-R score by 0.466 points on average comparing with 1–5 patients,
- >25 served patients: regression parameter is 0.379, which increases the BERNCA-R score by 0.379 points on average comparing with 1–5 patients,
- SWWS: regression parameter is −0.045, thus each point in SSP decreases the BERNCA-R score by 0.045 points on average (Table 5).

**Table 5:** Results of regression analysis.

**Feature**		**Parameter**	**95%CI**		***p***
Age	23–30 years	Ref.			
	31–40 years	0.127	−0.254	0.508	0.513
	41–50 years	0.023	−0.386	0.432	0.913
	>50 years	−0.026	−0.46	0.408	0.907
Seniority	0–5 years	Ref.			
	6–15 years	−0.111	−0.513	0.29	0.587
	16–20 years	−0.298	−0.718	0.123	0.166
	>20 years	−0.217	−0.627	0.193	0.299
Education	MSc in nursery/obstetrics (*N* = 83)	Ref.			
	Bachelor of nursery/obstetrics (*N* = 152)	0.081	−0.12	0.281	0.431
	Medical high school (*N* = 74)	−0.222	−0.487	0.042	0.1
	Medical college and “other” education (*N* = 220)	0.124	−0.077	0.326	0.227
Specialty	Yes	Ref.			
	No	−0.066	−0.21	0.079	0.373
Number of jobs	One	Ref.			
	Two or more	0.024	−0.114	0.163	0.731
Patient-to-nurse ratio	1–5 patients	Ref.			
	6–15 patients	0.675	0.404	0.945	<0.001^*^
	16–25 patients	0.466	0.17	0.763	0.002^*^
	>25 patients	0.379	0.053	0.704	0.023^*^
Marital status	In a relationship	Ref.			
	Single	0.135	−0.017	0.287	0.082
SWLS	[score]	0.014	0	0.027	0.059
SWWS	[score]	−0.045	−0.058	−0.033	<0.001^*^

*p**—**multifactorial linear regression*.

* *Statistically significant correlation (p <0.05)*.

The *R*^2^ coefficient for this model is 17.66%, which means that 17.66% of the BERNCA-R score variability was explained by the variables adopted in the model. The remaining 82.34% depends on the variables not included in the model and on random factors.

## Discussion

Care rationing seems to be both an increasingly recognized and relatively common practice in nursing care. Understanding the mechanisms of care rationing is very important (Scott et al., 2019). The purpose of this study was to assess the occurrence of care rationing among nurses working in surgical wards. An analysis of the obtained data demonstrated that the total mean BERNCA score is 1.53, which means that the frequency of care rationing by the respondents falls within the range between “never” and “rarely.” Similar results were also obtained by Jaworski et al. (2020), Schubert et al. (2013), and Uchmanowicz et al. during a validation study of the Polish version of the BERNCA questionnaire (Uchmanowicz et al., 2019a) and also the studies conducted among nurses working in a public hospital (Uchmanowicz et al., 2020a, 2021).

This study demonstrates that the activities most frequently rationed in patient care include analyzing the situation of individual patients and care plans at the beginning of the shift, assessment of the needs of newly admitted patients, administration of prescribed medication or infusion on time, setting up care plans for the patients, and providing emotional or psycho-social support to the patient, which is consistent with the results obtained by other authors (Schubert et al., 2013; Uchmanowicz et al., 2020a). In a study by Uchmanowicz et al. (2019a), the nurses declared that the most frequently rationed activities included activating or rehabilitating care, and due to inability to provide sufficient monitoring, they had to restrain confused patients or administer sedatives. Many authors also demonstrated that comfort talk with patients (Al-Kandari and Thomas, 2009; Ausserhofer et al., 2014; Bekker et al., 2015) and timelines of response to requests (Jones, 2014, 2015; Jarošová and Zeleníková, 2019; Gurková et al., 2020; Kalánková et al., 2020; Zeleníková et al., 2020) are the activities most frequently rationed when providing care to the patient.

This study reveals that nurses most frequently ration the activities related to the first and second stage of the caring process (diagnosing the bio-psycho-social condition of the patient and planning activities aimed at solving the problems of patients), which finally translates into lower quality of provided care and worse treatment outcomes for patients (Campagna et al., 2020). In contrast, the positive aspect is that, as has been demonstrated, the least frequently rationed activities include applying necessary disinfection measures and preparation of patients for planned tests or therapies, which is also confirmed by other authors (Schubert et al., 2013; Uchmanowicz et al., 2019a). The due performance of these activities enables the prevention of hospital-acquired infections and minimizes the risk of adverse events related to medical procedures or treatment.

Analysis of the obtained data demonstrated that the frequency of care rationing is significantly higher in persons working <5 years compared with nurses with seniority exceeding 20 years and among younger nurses (aged below 50 years) compared with persons aged above 50 years, which complies with the results obtained by Jarošová and Zeleníková (2019) and Al-Kandari et al. (Al-Kandari and Thomas, 2009).

It was also demonstrated that the factor of the patient-to-nurse ratio during a shift has a significant impact on the level of care rationing. Increasing the patient-to-nurse ratio results in more frequent non-completion of tasks planned during nursing care. Many authors (Schubert et al., 2013; Ausserhofer et al., 2014; Zhu et al., 2019) indicated that lower patient-to-nurse ratios are connected with lower levels of nursing care left undone.

An analysis of the obtained data revealed that the vast majority of the respondents (42.34%) declared a moderate level of satisfaction with life, which complies with the results achieved by other authors (Marilaf Caro et al., 2017; Kupcewicz et al., 2018; Piotrkowska et al., 2019; Lorber et al., 2020). While the study by Uchmanowicz et al. (2019b) demonstrated that nurses and midwives most frequently assess their level of life satisfaction as high. The difference in the results may be due to the characteristics of the study group, as the average age of the respondents in this study was significantly lower. While in the Iranian study, the vast majority of the respondents were definitely not satisfied with life (Yazdanshenas Ghazwin et al., 2016), which was affected by work-related factors (no satisfaction with remuneration and work environment) and associated with everyday emotions and stress. Practicing the profession of nurse is associated with experiencing strong stress and chronic fatigue, which clearly affects the perception and assessment of life satisfaction (Uchmanowicz et al., 2021).

In this study, no significant impact of the level of life satisfaction was on the level of rationing of nursing care (despite the result was borderline statistically significant), while a study by Uchmanowicz et al. (2021) demonstrated that nurses with a low or medium level of life satisfaction tend to leave nursing care unfinished more frequently compared with nurses who assess their level of life satisfaction as high.

Job satisfaction had a significant positive effect on the nurse-assessed quality of care (Boamah et al., 2017). An analysis of the material also demonstrated that the level of job satisfaction among the nurses in the study group was 18.5 points, which means that the nurses are neither satisfied nor unsatisfied with their job, which is consistent with the results obtained by other authors (Uchmanowicz et al., 2019b, 2020a; Jaworski et al., 2020). Aiken et al. (2013) revealed that the proportion of nurses unsatisfied with their job dramatically differ depending on the country of their work**—**in the Netherlands, only 11% of surveyed nurses reported no satisfaction with work, compared with 56% in Greece. The level of satisfaction with job should be continuously monitored by the management, since it was demonstrated that nurses unsatisfied with job ration the nursing care from 2.6 (White et al., 2019) to 3.4 (Clark and Lake, 2020) times more frequently compared with nurses reporting satisfaction with job, which was proved in studies conducted by other authors (Jones, 2014; Clark and Lake, 2020; Friganovic et al., 2020; Gurková et al., 2020; Uchmanowicz et al., 2020a,b; Zeleníková et al., 2020), as well as in this study. A low level of satisfaction with job also translates into a greater willingness to resign from work, and consequently, into staff shortages. Insufficient staff resources are a factor significantly affecting the level of rationing of nursing care (Alsubhi et al., 2020).

## Conclusions

The most frequently rationed care interventions identified in the implicit rationing approach were activities related to care plans. The assessment of the level of satisfaction with work and identification of affecting factors will enable reducing the occurrence of care rationing among the nurses working at the surgical wards in Polish hospitals.

## Limitation

This study has some limitations. Care workers might not report the actual level of care rationing. Also, the results are based on the self-reported measure of satisfaction with job and satisfaction with life. The final limitation is the use of a cross-sectional study design, which did not allow us to arrive at firm conclusions regarding the causality of predictors.

## Data Availability Statement

The datasets presented in this article are not readily available because personal data in the data set. Requests to access the datasets should be directed to aleksandra.koltuniuk@umed.wroc.pl.

## Ethics Statement

The study was carried out following the guidelines of the Declaration of Helsinki and Good Clinical Practice. Participation in the study was voluntary and anonymous. The study protocol was approved by the Bioethics Committee of Wrocław Medical University in Poland (permission no. KB-41/2019).

## Author Contributions

AK, IW, AM, KC, and IU: conceptualization, methodology, investigation, and writing—original draft preparation. AK and IW: formal analysis. AK: resources, data curation, writing—review and editing, and funding acquisition. IU: visualization. AK and IU: supervision. All authors have read and agreed to the published version of the manuscript.

## Conflict of Interest

The authors declare that the research was conducted in the absence of any commercial or financial relationships that could be construed as a potential conflict of interest.

## Publisher's Note

All claims expressed in this article are solely those of the authors and do not necessarily represent those of their affiliated organizations, or those of the publisher, the editors and the reviewers. Any product that may be evaluated in this article, or claim that may be made by its manufacturer, is not guaranteed or endorsed by the publisher.
